# Benign Duodenal Stricture Treated with Surgical Correction and Dietary Therapy in a Golden Retriever

**DOI:** 10.1155/2020/4283175

**Published:** 2020-04-06

**Authors:** John C. Rowe, Alice A. Huang, Jin Heo, Nolie K. Parnell, Adam J. Rudinsky

**Affiliations:** ^1^Department of Veterinary Clinical Sciences, The Ohio State University College of Veterinary Medicine, USA; ^2^Seattle Veterinary Specialists, USA; ^3^VCA West Coast Specialty and Emergency Hospital, USA; ^4^Department of Veterinary Clinical Sciences, Purdue University College of Veterinary Medicine, USA

## Abstract

A benign duodenal stricture is a well-documented condition of humans that has not been characterized in dogs. In this case report, the clinical, radiographic, ultrasonographic, endoscopic, surgical, and histopathologic findings of a single benign duodenal stricture in a Golden Retriever are reported. Definitive diagnosis was made possible with the utilization of esophagogastroduodenoscopy (EGD). Surgical correction of the stricture, paired with dietary therapy that utilized a highly digestible diet, resolved the clinical signs in the case reported. Several inciting causes were identified as possible drivers of stricture formation, including nonsteroidal anti-inflammatory drug (NSAID) administration, mucosal ulceration, traumatic injury, or inflammatory intestinal disease. A benign duodenal stricture should be considered an infrequent cause of intermittent, chronic gastrointestinal signs that may have a favorable outcome via surgical correction and dietary management.

## 1. Introduction

Duodenal strictures are rare in veterinary medicine and have not previously been reported in the canine species. They are most often identified as a sequela to another disease process or insult. The majority of the published cases have been attributed to ongoing inflammatory bowel disease, mucosal ulceration, NSAID usage, neoplastic malignancies, and pancreatitis [1–8]. These reports are derived from both human and veterinary subjects, and, since there is a paucity of evidence, trends in causality along species lines are difficult to determine. This report describes the clinical presentation, diagnosis, treatment, and long-term outcome in a dog diagnosed with a benign duodenal stricture.

## 2. Case Description

An 8.5 yr old, 30.7 kg male neutered Golden Retriever presented to Purdue University Veterinary Teaching Hospital (PUVTH), for referral evaluation of a 5-year history of intermittent vomiting, regurgitation, and loose stool. Review of primary care veterinarian medical records revealed that control of intermittent gastrointestinal signs was achieved with empirical metronidazole (16.2 mg/kg PO q 12) and metoclopramide (0.65 mg/kg PO q 12). The patient had also chronically received deracoxib (2.44 mg/kg PO q 24), sucralfate (1 gram PO q 12), tramadol (1.62 mg/kg PO q 12), and methocarbamol (16.2 mg/kg PO q 8) for pain management related to osteoarthritis and intervertebral disc disease. Six weeks prior to admission to PUVTH, the referring veterinarian discontinued the deracoxib when gastrointestinal clinical signs began to worsen. The patient otherwise had no past pertinent medical history, was up-to-date on core vaccinations, and received regular preventative healthcare which included an annual fecal analysis. The patient had a regular travel history to central Canada.

At presentation, the owner reported increasing lethargy and weight loss (5 kg) occurring over a six-week duration. The patient's appetite increased following a change from dry to canned food; however, no correlated changes in gastrointestinal clinical signs were reported. The patient had a complete blood count, biochemical profile, thyroid level, thoracic and abdominal radiographs, and a barium upper gastrointestinal tract contrast study prior to referral. No abnormalities were noted on laboratory analyses, thoracic radiographs, or abdominal radiographs. Barium contrast study revealed residual fluid retained within the stomach and suspected pyloric thickening.

Upon intake at PUVTH, physical examination was unremarkable. Initial diagnostic workup included the following: a complete blood count, blood chemistry, urinalysis, resting cortisol, cobalamin, folate, trypsin-like immunoreactivity (TLI), abdominal radiographs, and ultrasound. No abnormalities were identified with these first-line diagnostics. Thoracic radiographs revealed fluid opacity in the caudal esophagus consistent with either esophagitis, esophageal reflux, or less likely a mass lesion.

**Figure 1:**
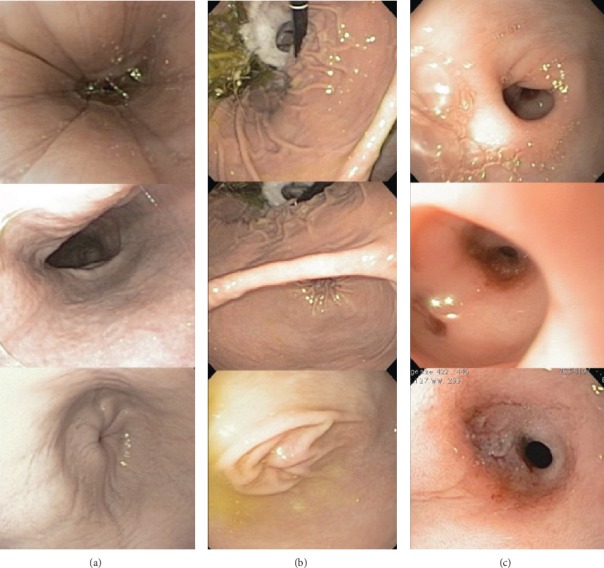
Images from the esophagogastroduodenoscopy performed at the time of diagnosis: (a) the esophagus, (b) stomach, and (c) proximal duodenal stricture.

Due to the persistence of gastrointestinal clinical signs and the lack of a definitive diagnosis on initial evaluation, the patient underwent endoscopic esophagogastroduodenoscopy (EGD). Static photographs are provided from performed EGD (Figure 1). Endoscopic evaluation of the esophagus revealed mild, circumferential, and shallow irregularities in the esophageal mucosa (Figure 1(a)). The gastric mucosa had a diffuse patchy erythema with white-colored mucosa noted in the fundus and pyloric antrum (Figure 1(b)). The pylorus was hypermotile and was easily traversed with the gastroscope. Immediately following passage through the pylorus, a circumferential dilatation between the pyloric opening and a duodenal stricture distal to the pyloric opening was noted. The stricture was estimated to be approximately 6 cm past the pylorus as measured by the endoscope. The 5 mm diameter opening of the stricture prevented further passage of the scope (Figure 1(c)). The visible duodenal mucosa had a slightly granular texture throughout. Biopsies were taken at the stricture site and proximal duodenum and the stomach. The histopathology results were consistent with fibrosis and mild lymphoplasmacytic inflammation.

**Figure 2:**
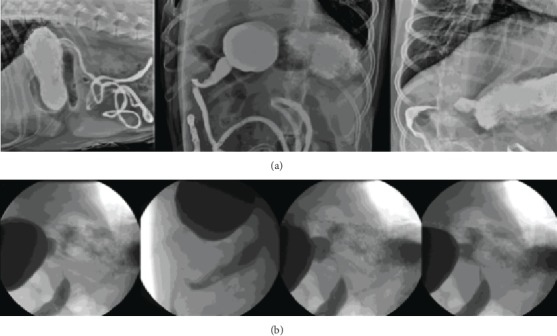
Contrast radiography (a) and fluoroscopy (b) of the proximal duodenal stricture.

Following EGD, a contrast (barium) upper gastrointestinal study was performed using static radiographs and fluoroscopy. Fluoroscopic evaluation revealed the barium passing through the pyloric canal, into the proximal duodenum, prior to identifying a stenotic segment of the proximal duodenum proximal to the cranial flexure. The barium pooled proximally to the stenotic area of the duodenum showing a “beak sign”. The stenotic areas and “beak-sign” are well visualized on the provided radiographs as well (Figures 2(a) and 2(b)).

Proximal gastrointestinal ultrasound following administration of the barium allowed for visualization of a focal narrowing of the lumen of the proximal duodenum aborad to the pylorus and orad to the cranial duodenal flexure. Orad to the narrowing, the focal widening of the proximal duodenum could be visualized. Summary of imaging findings were consistent with a proximal duodenal stricture.

Based on tentative diagnosis of the benign duodenal stricture, the dog underwent an abdominal exploratory surgery, stricture correction, and full-thickness gastrointestinal biopsies. A proximal duodenal stricture was confirmed at the time of surgery with a large amount of omental and mesenteric adhesions present. The biopsy and enterotomy taken at the stricture site were closed in a transverse fashion (4-0, PDS) to relieve the stricture and attempt to confirm a benign cause of stricture formation. Biopsies from the jejunum and ileum were also collected. Analysis of the biopsies revealed transmural granulomatous and lymphoplasmacytic enteritis at the site of stricture with focal mucosal ulceration and hyperplasia. Additional peritonitis was noted and attributed to a likely partial rupture at the stricture site. These findings were consistent with a benign, proximal duodenal stricture. The jejunal and ileal biopsy findings were unremarkable.

The dog recovered uneventfully from surgery and at the two-week postoperative recheck was free of clinical signs. Initially, it was planned that after the full-thickness biopsies of the stricture were obtained, the dog would return for a more aggressive surgical correction (e.g., Billroth 1 for surgical margins if neoplastic) if indicated. However, based on the observed clinical response, it was deemed unnecessary. Resolution of clinical signs was achieved for 18 months postoperatively with dietary therapy utilizing a highly digestible diet (Hill's Prescription Diet i/d Digestive Care); at which time, the dog was euthanized following acute collapse, hemoabdomen, and a suspected hemangiosarcoma based on masses identified by abdominal ultrasound (liver and spleen) and cytology (liver).

## 3. Discussion

This report describes a case of a benign duodenal stricture in a dog. In humans and other veterinary species, duodenal strictures appear to be an uncommon phenomenon and often a sequela to other disease processes. A medical literature search reveals most cases of duodenal strictures have been reported in association with inflammatory bowel disease, mucosal ulceration, NSAID usage, neoplastic malignancies, and pancreatitis [1–8]. These reports are derived from both human and veterinary subjects; however, as there is a paucity of evidence in any particular species, trends in causality along species lines are difficult to determine.

In humans, although uncommon, duodenal strictures are a complication reported secondary to Crohn's disease [9]. In those patients, an underlying and inciting inflammatory process precedes and directly mediates a fibrotic response leading to ultimate stricture formation [10]. In the case presented, it is possible that there was a chronic enteropathy present, such as inflammatory bowel disease (IBD), which eventually led to the development of a duodenal stricture. The chronic history of gastrointestinal signs and lymphoplasmacytic inflammation identified on biopsies was consistent with a diagnosis of chronic enteropathy. Clinical signs resolved following surgical correction of the stricture area, and the fact that the primary chronic management tool, long-term dietary therapy with an easily digestible diet, prevented recurrence of intermittent clinical signs is potentially supportive of mild chronic enteropathy in the patient described here.

Alternatively, the prevalence of chronic enteropathies in the canine population is significant and there are no available reports demonstrating the phenomenon of strictures developing secondary to inflammation as seen in people. In people, it is typically very chronic and severe cases that develop gastrointestinal strictures related to IBD. The dog reported here had milder, intermittent signs, not matching the expected severity which would cause a stricture in other species. Furthermore, the biopsies, which were consistent with a chronic enteropathy, were mostly showing a mild inflammatory reaction and only noted in those taken at the site of stricture. All additional biopsies along the remaining length of the intestinal tract were not indicative of significant widespread lymphoplasmacytic infiltrative inflammation. All biopsy reports were characterized based on the attending pathologist's interpretation of the tissues but were not evaluated using WSAVA histopathology guidelines. This lack of standardization makes assessment of the degree of inflammation challenging in these samples. Therefore, the biopsies are more likely simply indicative of focal lymphoplasmacytic intestinal inflammation at the site of stricture and very unlikely to be related to a focal form of a chronic enteropathy such as inflammatory bowel disease. Based on this information, the clinical response seen following surgery is most likely indicative of removing the stricture and its role causing a partial obstruction resulting in the clinical signs.

Another potential explanation for the duodenal stricture in the dog reported here is the prolonged history of an upper-end maintenance dose (2.44 mg/kg) of the NSAID deracoxib. When deracoxib was given in a randomized, placebo-controlled trial to healthy dogs at a 1.5 mg/kg dose, the gastric mucosa was not significantly different than dogs receiving a placebo; however, medications were only administered for 28 days and the impact of chronic therapy is unknown [11]. Additionally, a case series of three dogs receiving postoperative deracoxib (2.5-3 mg/kg) all developed duodenal perforation and subsequent peritonitis at the same location just orad to the major duodenal papilla seen in the dog described here [12]. Deracoxib, like many other NSAIDs, is excreted largely through the liver and undergoes enterohepatic recycling, predisposing this precise location to increased doses of the medication continuously as bile is released [13, 14].

The mechanism of ulceration associated with NSAID use is multifactorial. The common result of these mechanisms is damage and injury to the gastrointestinal mucosa [15]. The biopsy collected from the site of stricture, which showed mucosal ulceration in conjunction with the focal nature of the lesion, suggests that there was a potential for ulcer creation at the duodenal mucosa in this patient. Further, a partial rupture, as suggested by the peritonitis at the biopsy site, could be a logical driver for the fibrosis and cellular response [10]. Therefore, there is potential that the cause of the duodenal stricture in this patient was ulceration of the duodenal mucosa in response to extended high maintenance dosing of a NSAID.

Within other veterinary species, duodenal strictures have been reported, with the highest frequency occurring in foals. In these veterinary reports, the clinical presentation and suspected mechanisms revolve around gastrointestinal mucosal ulceration prior to stricture formation. In foals, specifically, concerns related to NSAID involvement has prompted the recommendation that treatment and prophylaxis be centered around mitigating overuse of NSAIDs and coadministration of antiulcer medication [2]. Within the human medical population, NSAID usage can cause endoscopically evident small-intestinal mucosal injury and can lead to stricture formation [16, 17]. In a study of 21 long-term NSAID users and 20 control patients, 71% of NSAID users had endoscopic evidence of mucosal damage in the intestine compared to 10% of control patients [16]. A separate report exists of four human patients with small intestinal stricture formation thought to be secondary to chronic NSAID usage [17]. Thus, it has been well established in human medical literature that chronic NSAID usage in isolated cases can result in a similar pathogenesis of intestinal mucosal ulceration, stricture formation, and even perforation [18].

In humans, many other causes of gastrointestinal tract ulceration (peptic ulcer disease), infection (*Helicobacter pylori*, tuberculosis), caustic injury, traumatic injuries, and other diseases (neoplastic malignancies, pancreatitis) are conditions that may possibly cause duodenal stricture [3, 4]. The single canine case report of duodenal stricture was shown to develop a postoperative duodenal stricture following a peritoneal draining procedure for known peritonitis [5]. In this instance, there was a presumed injury to the duodenum from caustic, inflammatory, and traumatic injury that resulted to stricture formation. Reports of these mechanisms causing duodenal strictures are published in people as well [19, 20]. An additional traumatic injury consideration would be the generation of focal trauma from a previous intestinal foreign body similar to what is more commonly seen with esophageal injury in dogs [21]. There were no reported historical exposures to foreign bodies or other insults, but these potential explanations cannot be excluded.

Based on the dog described here having a long postdiagnosis survival, no evidence of infection or neoplasia on biopsy, and lacking a traumatic event or caustic ingestion, these differential diagnoses seem unlikely. Benign duodenal strictures have been reported in people but have not yet been reported in dogs [19, 22]. This remains a potential reason for stricture formation in this dog.

Ultimately, this patient was managed with dietary therapy and surgical resection; however, duodenal strictures in people are also managed with balloon dilation and stenting which may have been viable alternative approaches in this case [22]. In this case, a highly digestible diet was ideal for maintaining the resolution of gastrointestinal signs following surgery. A highly digestible diet was the ideal choice for this dog given any possible reduction in motility or functionality of the proximal duodenum following surgical correction. Ensuring that the particulate matter of the diet was minimized was crucial for this patient, so a highly digestible diet with lower residue was selected as the principal postoperative therapy. Further, since mild evidence supportive of some degree of IBD had been seen on histopathology, a highly digestible diet was again indicated [23]. In a randomized diet study, a highly digestible diet was able to mitigate IBD clinical signs in all dogs (*n* = 12) for three months, although most had reemergence of clinical signs at six- and 12-month follow-up [24]. Again, since the dog presented in this particular case did not have reemergence of gastrointestinal signs while maintained on the highly digestible diet, it is less likely that this dog had profound IBD capable of initiating stricture formation in the first place. It may also be worth mentioning that in human Crohn's disease patients, dietary therapy is an emerging cornerstone of combination therapy relying upon either partial enteral nutrition or specific elimination diets, both of which provide highly digestible nutrition among other dietary attributes [25].

Another interesting aspect of the case reported here is that many initial imaging modalities utilized in the primary care diagnostics workup were unable to diagnose the duodenal stricture as the reason for worsening gastrointestinal tract signs. Advanced imaging techniques utilized initially were unable to precisely detect the lesion in this case. Ultimately, it was only with the additional step of EGD that it was possible to diagnose a stricture in this patient and later on modification to advanced imaging (barium study with fluoroscopy). While the authors recognize EGD is not a feasible or warranted diagnostic modality for all gastrointestinal workups, this case underscores the important role endoscopy can play when advanced imaging studies remain inconclusive in a patient not responding to medical management and dietary therapy.

In summary, this report details a benign duodenal stricture in a dog presenting with gastrointestinal clinical signs. Surgical correction of the stricture and maintenance therapy utilizing a highly digestible diet completely resolved the gastrointestinal signs in this dog. While many drivers of stricture formation have been identified in the human and veterinary medical literature, pinning down a specific mechanism in this case is not clear. The most likely contributor might have been chronic deracoxib NSAID administration; however, an intestinal inflammatory process, idiopathic local mucosal ulceration, or a past pancreatitis event are also considerations.
